# Neuropsychiatric Sequelae of Chronic Rhinosinusitis: A Retrospective Cohort Study Using a Multi-institutional Electronic Health Records Network

**DOI:** 10.7759/cureus.100862

**Published:** 2026-01-05

**Authors:** Ioannis Zerefos, Zachary Li, Katherine M Napalinga

**Affiliations:** 1 Psychiatry, Reading Hospital, Tower Health, West Reading, USA; 2 Psychiatry, Drexel University College of Medicine, Philadelphia, USA

**Keywords:** anxiety, bipolar disorder, chronic rhinosinusitis, depression, inflammation, nasal polyps, neuropsychiatry, psychosis, schizophrenia, suicidal ideation

## Abstract

Background: Chronic rhinosinusitis (CRS), a common inflammatory disorder of the sinonasal mucosa, is associated with significant morbidity and impaired quality of life. Emerging evidence suggests a link between systemic inflammation and neuropsychiatric disorders, including depression and suicide risk. This study examines whether phenotypic differences in CRS, specifically CRS with nasal polyps (CRSwNP) and CRS without nasal polyps (CRSsNP), are associated with differing psychiatric outcomes.

Objective: The objective of this study was to evaluate whether CRSwNP and CRSsNP are differentially associated with suicidality and psychiatric disorders.

Methods: A retrospective cohort analysis was conducted using the TriNetX research network. Adults diagnosed with CRSwNP or CRSsNP between 2010 and 2023 were identified via the International Statistical Classification of Diseases and Related Health Problems, 10th Revision (ICD-10) codes. Propensity score matching was used to control for demographics and comorbidities, yielding two matched cohorts (n=130,769 each). Primary outcomes included suicidal ideation and suicide attempts. Secondary outcomes included depression, generalized anxiety disorder, bipolar disorder, schizophrenia, schizoaffective disorder, and psychosis. Risk differences, risk ratios, and odds ratios were calculated with 95% confidence intervals.

Results: Patients with CRSwNP demonstrated significantly lower risks of suicidal ideation (risk ratio (RR) 0.66) and suicide attempt (RR 0.63) compared to those with CRSsNP (all p<0.001). CRSwNP was also associated with lower risks of developing depression (RR 0.80), generalized anxiety disorder (RR 0.75), bipolar disorder (RR 0.72), schizophrenia (RR 0.63), schizoaffective disorder (RR 0.63), and psychosis (RR 0.64), although absolute risk differences were small.

Conclusion: CRSwNP is associated with a significantly lower risk of several psychiatric disorders compared to CRSsNP, potentially due to differences in underlying inflammatory pathways or treatment effects. These findings support the integration of psychiatric screening in CRS management and warrant further investigation into immune modulation as a potential strategy to reduce neuropsychiatric risk.

## Introduction

Chronic rhinosinusitis (CRS) is a prevalent inflammatory condition of the paranasal sinuses and nasal mucosa, affecting approximately 10-12% of the global adult population and significantly diminishing quality of life [1]. CRS is broadly categorized into two phenotypes: CRS with nasal polyps (CRSwNP) and CRS without nasal polyps (CRSsNP). These subtypes exhibit distinct immunologic profiles, with CRSwNP typically characterized by a type 2 inflammatory response dominated by interleukins (ILs), IL-4, IL-5, and IL-13, leading to eosinophilic inflammation, whereas CRSsNP is more often associated with type 1 or type 3 inflammation and neutrophilic infiltration [2,3].

Suicide remains a pressing public health crisis, ranking as the 11th leading cause of death in the United States in 2023, with more than 49,000 deaths and an estimated 1.5 million attempts [4]. Suicide disproportionately affects vulnerable populations, including adolescents, young adults, and middle-aged men, and carries a profound societal and economic toll [5]. Importantly, suicide is preventable, underscoring the need to identify medical and psychiatric risk factors that may increase vulnerability.

A growing body of evidence suggests a significant link between immune dysregulation, chronic low-grade inflammation, and the pathophysiology of major depressive disorder (MDD) and suicidality [6-8]. Pro-inflammatory cytokines such as IL-6, tumor necrosis factor-alpha (TNF-α), and C-reactive protein (CRP) are consistently elevated in individuals with MDD. These mediators can cross the blood-brain barrier and alter serotonin, dopamine, and glutamate signaling through the kynurenine pathway, contributing to neuroinflammation, impaired neuroplasticity, and dysregulation of the hypothalamic-pituitary-adrenal axis-all processes implicated in suicidal behavior [9,10]. The burden of CRS extends beyond the sinonasal system, with increasing recognition of its neuropsychiatric implications. Persistent symptoms such as facial pain, sleep disturbance, and anosmia impair health-related quality of life and are independently linked to higher rates of depressive symptoms [11]. Systemic cytokines elevated in CRS, including IL-6, TNF-α, and IL-1β, overlap with pathways implicated in MDD and suicidality [6]. Furthermore, olfactory dysfunction common in CRS may disrupt limbic connectivity, a critical hub for emotional regulation, thereby predisposing to depression [12]. Prior research demonstrates that individuals with CRS have an increased risk of suicidal ideation and behaviors even after adjusting for comorbid psychiatric and demographic factors [13].

Subgroup differences further suggest that CRSsNP may confer a greater psychiatric burden than CRSwNP, with higher reported rates of depression and anxiety [14]. Symptomatology also differs, as CRSwNP patients more frequently experience nasal congestion and olfactory loss, whereas CRSsNP patients report more severe pain, which may contribute to mood disturbance [11]. Whether these clinical and inflammatory differences extend to suicidality and severe psychiatric disorders, however, remains unknown. Beyond depression and anxiety, chronic immune activation has also been implicated in psychotic disorders, including schizophrenia and schizoaffective disorder, as well as in bipolar disorder [15]. These associations support a broader neuroimmune model of psychiatric illness, raising the possibility that CRS phenotypes may differentially influence risk for serious mental illness. The present study, therefore, aims to investigate how CRS subtypes are associated with neuropsychiatric morbidity, with particular focus on suicidality, mood disorders, and severe mental illness.

## Materials and methods

This retrospective cohort study used de-identified electronic health record data from the TriNetX research network (TriNetX LLC, Cambridge, Massachusetts, United States), a global federated health research platform that aggregates data from participating healthcare organizations. Only aggregated, de-identified data were used in compliance with the Health Insurance Portability and Accountability Act (HIPAA), and no patient-level identifiers were accessed. Over 100 healthcare organizations, primarily across the United States, were utilized.

Study population

Two cohorts were identified: adults (age ≥18) diagnosed with CRS between January 1, 2010, and December 31, 2023. Patients were classified using the International Statistical Classification of Diseases and Related Health Problems, 10th Revision (ICD-10) codes into CRSwNP (J33 + J32) and CRSsNP (J32 only). The index event was the first occurrence of the relevant diagnostic code. Patients whose index event occurred more than 20 years prior to analysis were excluded.

**Figure 1:**
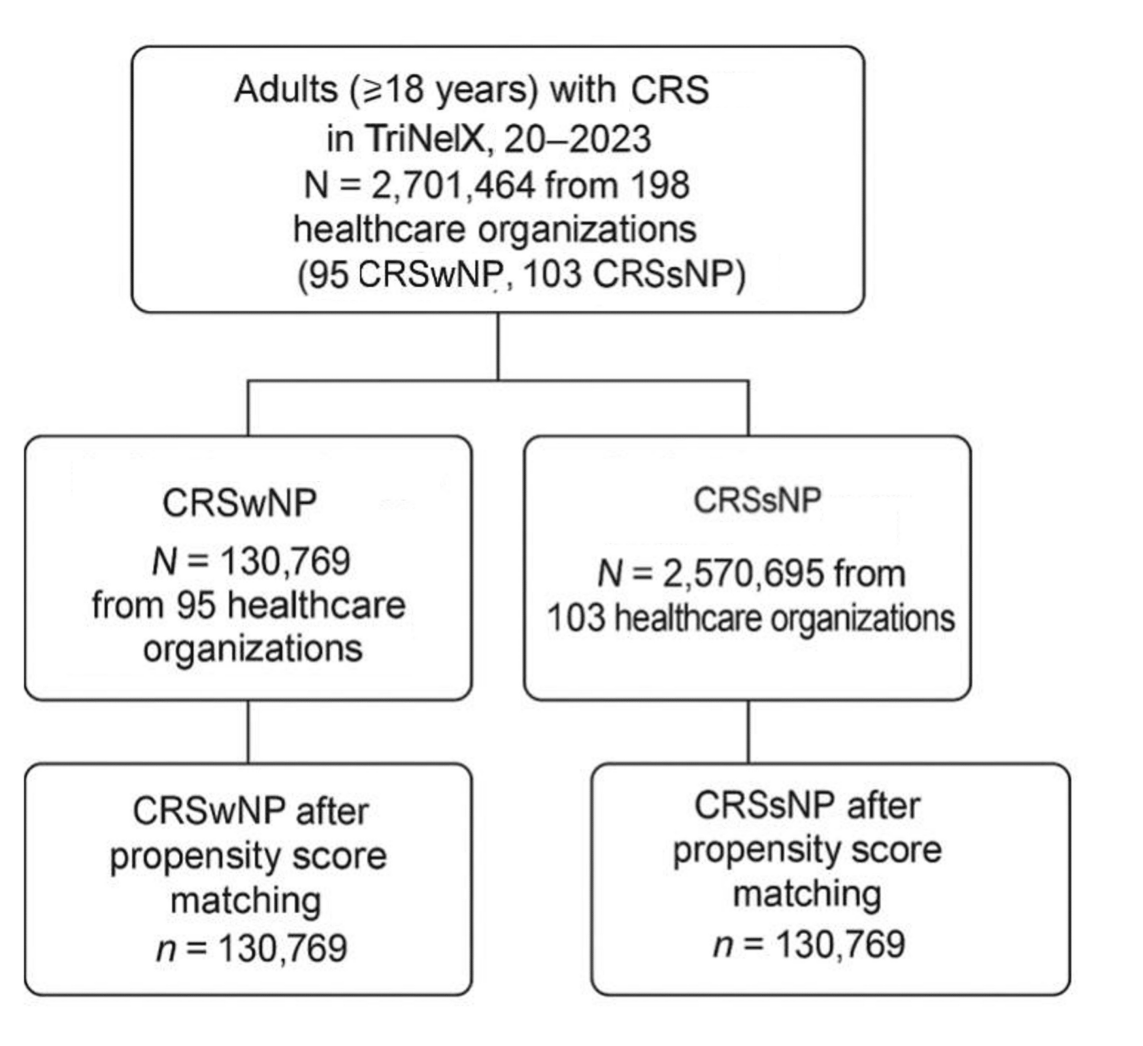
Flowchart of cohort construction from the TriNetX network for CRSwNP and CRSsNP CRSwNP: chronic rhinosinusitis with nasal polyps; CRSsNP: chronic rhinosinusitis sans (without) nasal polyps; CRS: chronic rhinosinusitis

The CRSwNP and CRSsNP cohorts initially included 130,769 patients from 95 healthcare organizations and 2,570,695 patients from 103 healthcare organizations, respectively, for a total of 2,701,464 adults with CRS. Propensity score matching was performed using a nearest-neighbor algorithm with a caliper of 0.1 to balance age, sex, race, body mass index (BMI), and nicotine dependence, resulting in two cohorts of equal size (n = 130,769 per group). A flowchart of cohort construction is provided in Figure 1.

Outcomes

The primary outcomes of interest were suicidal ideation (ICD-10-CM: R45.851) and suicide attempt (ICD-10-CM: T14.91). Secondary outcomes included depression, defined as either major depressive episodes (MDE) or MDD (ICD-10-CM: F32, F33), psychosis (ICD-10-CM: F29), schizoaffective disorder (ICD-10-CM: F25), schizophrenia (ICD-10-CM: F20), bipolar disorder (ICD-10-CM: F31), and generalized anxiety disorder (GAD) (ICD-10-CM: F41.1). Outcomes were measured beginning one day after the index event, with no set endpoint. Patients with a documented outcome prior to the index event were not excluded from analysis, which limits the ability to infer temporal or causal relationships.

Data analysis

Risk analyses were performed for each outcome. Results were reported as risk ratios (RR), risk differences (RD), and odds ratios (OR), each with 95% confidence intervals (CIs), and, along with p-values, were calculated by the TriNetX software. All analyses were conducted using the TriNetX Analytics platform, where between-group differences in categorical outcomes were evaluated using chi-square tests applied to two-by-two contingency tables, as implemented in the TriNetX analytics platform. Continuous baseline variables were compared using independent-samples t-tests. 

## Results

After propensity score matching, both the CRSwNP and CRSsNP cohorts consisted of 130,769 patients with comparable demographic characteristics. The mean follow-up time was 1,861.52 days (median 1,564 days) for CRSwNP and 2,027.42 days (median 1,795 days) for CRSsNP.

As summarized in Table 1, patients with CRSsNP demonstrated a consistently higher prevalence of every psychiatric outcome evaluated. Individuals with CRSwNP had significantly lower rates of suicidal ideation, suicide attempts, depression, anxiety, bipolar disorder, psychosis, schizophrenia, and schizoaffective disorder. This pattern was reflected across all effect measures, with risk ratios generally ranging from approximately 0.63 to 0.80.

**Table 1:** Summary of results for each psychiatric outcome Baseline characteristics and psychiatric outcomes in patients with chronic rhinosinusitis with nasal polyps (CRSwNP) compared to those without nasal polyps (CRSsNP) after propensity score matching. Values are presented as n (%). p-values were calculated using chi-square tests for categorical variables and independent-samples t-tests for continuous variables, as implemented in the TriNetX Analytics platform.

Outcome	CSwNP (%)	CRSsNP (%)	Risk Ratio (95% CI)	Odds Ratio (95% CI)	p-value
Suicidal ideation	0.9	1.4	0.66 (0.62–0.72)	0.66 (0.61–0.71)	<0.001
Suicide attempt	0.1	0.1	0.63 (0.50–0.80)	0.64 (0.50–0.80)	<0.001
Depression	17.2	21.6	0.80 (0.78–0.81)	0.75 (0.74–0.77)	<0.001
Generalized anxiety disorder	6.8	9.1	0.75 (0.74–0.77)	0.74 (0.72–0.76)	<0.001
Bipolar disorder	1.7	2.4	0.72 (0.68–0.76)	0.71 (0.68–0.75)	<0.001
Psychosis (unspecified)	0.5	0.7	0.64 (0.58–0.71)	0.64 (0.57–0.70)	<0.001
Schizophrenia	0.3	0.5	0.63 (0.56–0.71)	0.63 (0.56–0.71)	<0.001
Schizoaffective disorder	0.2	0.4	0.63 (0.55–0.72)	0.63 (0.54–0.72)	<0.001

Depression and GAD represented the most common diagnoses and showed the largest absolute differences between cohorts, with patients with CRSwNP exhibiting markedly reduced risk compared to those with CRSsNP. In contrast, the psychotic disorders, schizophrenia, schizoaffective disorder, and unspecified psychosis, were less common overall but demonstrated the strongest relative protection in CRSwNP.

## Discussion

This large, multi-institutional cohort study found that CRSsNP was associated with significantly higher risk of suicidality, suicide attempts, depression, anxiety, bipolar disorder, psychosis, schizophrenia, and schizoaffective disorders compared to CRSwNP. Although the absolute risk differences were modest, the associations were robust across outcomes, underscoring that the CRS phenotype is an important factor in psychiatric vulnerability.

**Figure 2:**
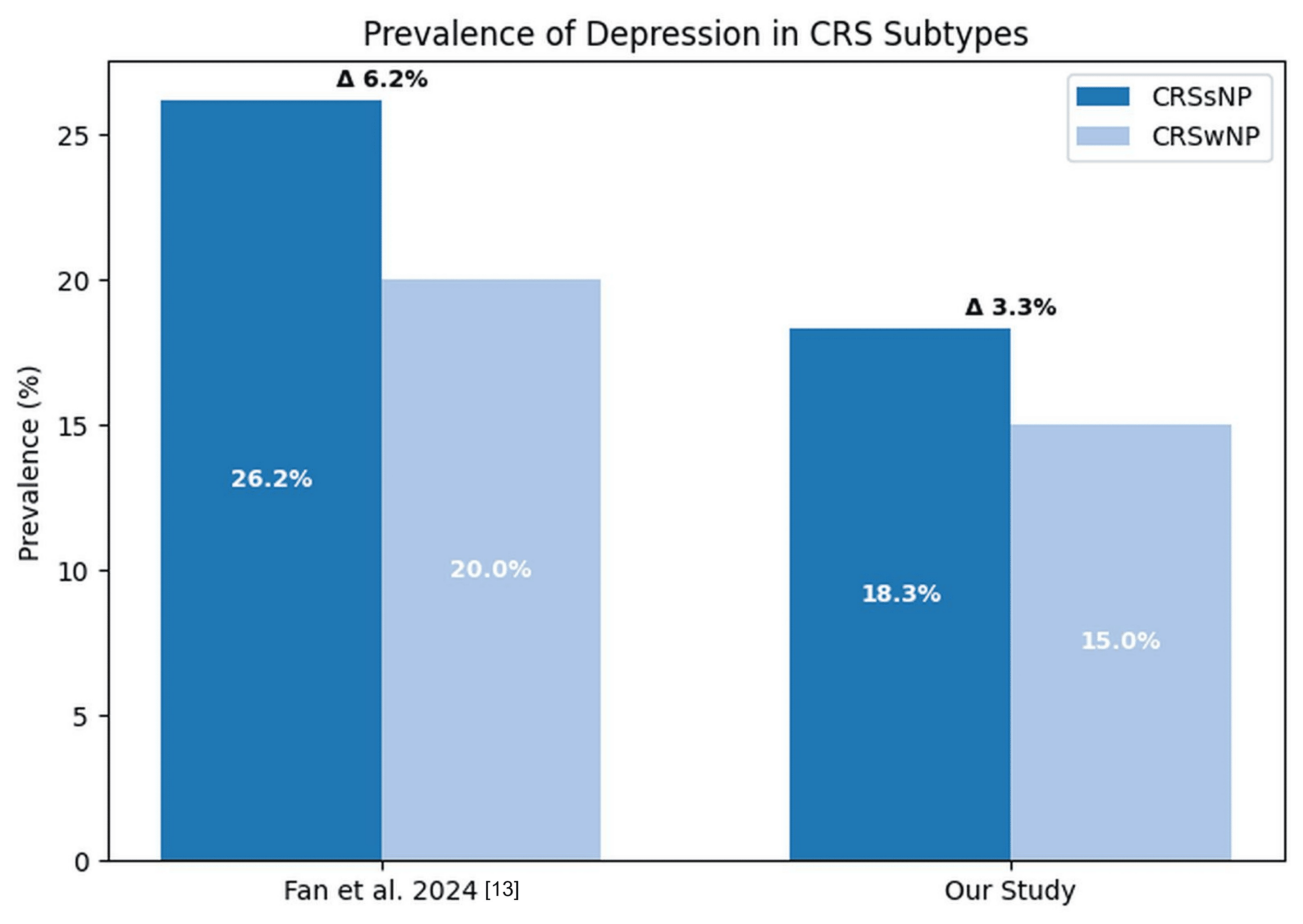
Comparison of the prevalence of depression in CRSwNP and CRSsNP cohorts between two studies Comparison between the current study and Fan et al., 2024 [13]. Both studies show consistently higher prevalence of depression in CRSsNP patients, although absolute rates differ. Δ signifies the prevalence percentage difference between the polyp and no polyp group in each study CRSwNP: chronic rhinosinusitis with nasal polyps; CRSsNP: chronic rhinosinusitis sans (without) nasal polyps; CRS: chronic rhinosinusitis

The association between CRS and neuropsychiatric conditions such as depression and anxiety has been well documented, with patients with CRS showing a significantly higher likelihood of developing these disorders. Prior studies further suggest that depressive symptoms and anxiety are more prevalent in CRSsNP compared to CRSwNP, pointing to potential differences in underlying disease pathways that may predispose patients to neuropsychiatric morbidity and symptom severity [13]. Our findings align with this study, demonstrating a significantly lower risk of developing depressive episodes and anxiety disorders in patients with CRSwNP relative to those with CRSsNP (Figure 2).

To our knowledge, no prior study has directly compared CRS subtypes in relation to suicidal ideation, suicide attempts, or the development of other major neuropsychiatric conditions. In this study, we observed a consistently lower risk of both suicidal ideation and suicide attempts among patients with CRSwNP compared to those with CRSsNP. This finding is particularly noteworthy given the established association between chronic systemic inflammation and heightened psychiatric risk. The observed differences underscore the possibility of distinct biological mechanisms across CRS phenotypes that extend beyond mood and anxiety disorders to influence a broader range of mental health outcomes.

CRS, irrespective of nasal polyps, is marked by persistent inflammation, but the immunologic profiles differ: CRSwNP is dominated by type 2 inflammation (IL-4, IL-5, IL-13, eosinophils), whereas CRSsNP is more often driven by type 1 and 3 responses with neutrophilic infiltration. Our data, while not causal, does speculate that this distinction may be clinically relevant, as immune dysregulation and low-grade systemic inflammation have been implicated in MDD and suicidality as per prior literature [6-10].

In addition, this discussion is intended to be hypothesis-generating rather than mechanistic, as inflammatory biomarkers, neuroimaging measures, and treatment exposures were not directly assessed in this study. However, emerging neuroimaging studies have come to support an inflammation-brain link. Sinonasal inflammation has been associated with altered connectivity in salience, default-mode, and frontoparietal networks [16], while salience network enlargement has been observed in MDD, particularly in the anterior cingulate and insula [17]. These networks are critical for emotion regulation and self-referential processing, providing a mechanistic bridge between peripheral inflammation and mood disorders.

These inflammatory mediators have also been shown to shift tryptophan metabolism toward neurotoxic metabolites such as quinolinic acid, implicating the kynurenine pathway in mood disorders and suicidality [15,18]. Beyond depression, chronic immune activation has also been associated with psychotic and bipolar disorders [19,20], which were likewise elevated in our CRSsNP cohort. Together, these findings suggest that CRS-related inflammation may disrupt large-scale brain circuits underlying a spectrum of psychiatric outcomes, from mood and anxiety to suicidality and psychosis, although further research validating this link will be required. 

Beyond mood and anxiety disorders, our findings also highlight increased risks of bipolar disorder, schizophrenia, and psychosis in CRSsNP compared to CRSwNP. Notably, among the psychiatric outcomes examined within our study, depression and anxiety demonstrated the largest absolute risk reductions among the psychiatric outcomes examined, reflecting their higher baseline prevalence and corresponding to a greater number of affected patients. However, schizophrenia and schizoaffective disorders represent the strongest relative protection (RR ~0.63) with an overall smaller burden due to the rarity of diagnosis compared to MDD/GAD.

Functionally, immune dysregulation has long been implicated in the pathophysiology of these disorders. Meta-analyses show that patients with schizophrenia and bipolar disorder have consistently elevated peripheral cytokines, including IL-6, TNF-α, and IL-1β, as well as dysregulated T-cell function [21-24]. Such inflammatory changes overlap with those observed in CRS, particularly the type 1 and type 3-driven responses of CRSsNP. In addition, microglial activation, HPA (hypothalamic-pituitary-adrenal) axis alterations, and complement cascade abnormalities have been linked to aberrant synaptic pruning and cortical connectivity disruptions in psychosis and bipolar disorder [25]. Taken together, these findings suggest that the systemic inflammatory environment in CRSsNP may exacerbate neuroimmune pathways relevant not only to depression and suicidality but also to the development of other serious mental illnesses.

Treatments for CRSwNP primarily aim to reduce inflammation and improve sinonasal symptoms. These treatments range from topical and systemic corticosteroids to more targeted biological therapies. Systemic corticosteroids are broad anti-inflammatory agents that can reduce general inflammation, including type 2 inflammation [1]. Biologic therapies, such as anti-IL-4Rα (dupilumab), anti-IL-5 (mepolizumab, reslizumab), or anti-IgE (omalizumab), specifically target key mediators of type 2 inflammation characteristic of CRSwNP [26-29]. By reducing systemic inflammation, particularly via biologics, psychiatric outcomes could theoretically improve. If chronic peripheral inflammation contributes to neurochemical changes observed in depression and suicidality, then dampening this inflammation may mitigate risk.

Olfactory dysfunction, common in CRS, may also exacerbate vulnerability. Studies show that impaired olfaction contributes to mood disturbance through disruption of limbic connectivity and emotion regulation circuits [30]. This further supports a multimodal pathway linking sinonasal inflammation, sensory dysfunction, and psychiatric burden.

The present findings raise important questions about the role of type 2 inflammation in neuropsychiatric health. Future studies should evaluate whether therapies targeting type 2 pathways, such as dupilumab, mepolizumab, omalizumab, or corticosteroids, confer psychiatric benefits in addition to sinonasal disease control. Our observation of lower depression and anxiety in CRSwNP parallels data from a large real-world atopic dermatitis cohort, where dupilumab users had significantly lower hazards of depressive and anxiety disorders compared with conventional therapy [28]. Whether type 2 inflammation itself is protective or whether treatment effects drive these associations remains unclear. Given that type 2 pathways are central to asthma, atopic dermatitis, eosinophilic esophagitis, and allergic rhinitis, cross-disease investigations may help determine whether reduced psychiatric risk reflects disease-specific or systemic immune mechanisms. Longitudinal, biomarker-driven, and neuroimaging studies are needed to disentangle these relationships.

This study has several limitations. First, the retrospective design and reliance on electronic health records introduce potential biases related to diagnostic coding accuracy and unmeasured confounding. Although the large sample size strengthens generalizability, residual confounders such as socioeconomic status, medication exposure, and comorbid allergic or autoimmune conditions could not be fully controlled. Second, psychiatric diagnoses were based on ICD-10 coding rather than structured clinical interviews, which may lead to diagnostic misclassification. Third, a clear causal and temporal relationship between CRS subtype and psychiatric outcomes cannot be established in this cross-sectional framework. We also acknowledge that patients with a documented outcome prior to the index event were not excluded from the study. Our objective was to compare the overall psychiatric burden between CRS phenotypes, rather than to establish temporal causality. From a clinical perspective, the presence of psychiatric comorbidity, regardless of onset, has important implications for screening, management, and health care utilization, and this is an acknowledged limitation. Finally, the use of deidentified multi-institutional data precludes verification of clinical details, such as disease severity, corticosteroid use, or biologic exposure. Future prospective, biomarker-driven studies are needed to confirm these associations and clarify causal pathways. Although propensity score matching was used to balance measured covariates, residual confounding remains possible, particularly from unmeasured factors such as medication exposure (e.g., corticosteroids or biologics), CRS disease severity, symptom burden, and treatment history, which were not directly captured in the dataset.

## Conclusions

This study reveals a significant association between CRSsNP and an increased risk of multiple psychiatric disorders, including suicidal ideation, suicide attempts, depression, bipolar disorder, psychosis, schizophrenia, and schizoaffective disorder, when compared to patients with CRSwNP. While no direct correlation can be drawn from the nature of this study, we speculate that treatment with anti-inflammatory or immunosuppressive medications in patients with CRSwNP may contribute to the observed associations; however, medication exposure was not assessed in this dataset, and this interpretation remains hypothetical and hypothesis-generating rather than causal. Future research should aim to further explore this potential mechanism. These findings emphasize the importance of screening for mental health conditions in patients with CRS and support further investigation into the role of inflammation and targeted treatment in mitigating psychiatric comorbidities of these diagnoses.
